# Vascular changes on fluorescein angiography of premature infants with low risk of retinopathy of prematurity after high oxygen exposure

**DOI:** 10.1186/s40942-016-0055-6

**Published:** 2017-01-16

**Authors:** Maria Ana Martinez-Castellanos, Raul Velez-Montoya, Kenneth Price, Andree Henaine-Berra, Gerardo García-Aguirre, Virgilio Morales-Canton, Linda Alejandra Cernichiaro-Espinosa

**Affiliations:** 1 Retinal Department, Asociación para Evitar la Ceguera en Mexico, “Hospital Luis Sanchez Bulnes”, IAP, Vicente Garcia Torres No. 46 Coyoacán, 04030 Mexico City, D.F. Mexico; 2 New York University School of Medicine, New York University, New York, NY 10012 USA

**Keywords:** Retinopathy of prematurity, Angiography, Oxygen, Vasculogenesis, Toxicity

## Abstract

**Purpose:**

To describe a wide array of peripheral vascular changes using fluorescein angiography in preterm neonates, without high risk characteristics for developing retinopathy of prematurity, that were exposed to high oxygen concentration.

**Methods:**

Retrospective, two center, case series. Newborns at two different hospitals with ≥1500 g or gestational age of ≥32 weeks, fluorescein angiography performed, and with high oxygen exposure without adequate control were included.

**Results:**

294 infants diagnosed with ROP were analyzed. Only 28 eyes from 14 patients with peripheral vascular abnormalities in older and heavier babies were included. Two distinct type of peripheral vascular changes were observed: group 1 or non-proliferative: areas of capillary non-perfusion along with widespread arteriovenous shunting between adjacent primary vessels, tortuosity of primary vessels, abnormal budding of tertiary vessels and capillaries, abnormal capillary tufts and absence of foveal avascular zone; group 2 or proliferative: all of the characteristics of group 1 plus leakage of dye from the boundary between perfused and non-perfused retina and/or optic disc.

**Conclusion:**

Peripheral vascular abnormalities different from retinopathy of prematurity are observed in older than 32 weeks of gestational age, and heavier than 1500 g babies. This makes the authors classify these patients as having a disease caused solely by oxygen dysregulation at the neonatal intensive care unit similarly to the oxygen induced retinopathy in experimental studies.

## Background

Retinopathy of prematurity (ROP) is a well-known complication of preterm births, which currently represent about 10% of all births worldwide [1–3]. It remains as one of the leading causes of child blindness and visual disability despite aggressive governmental surveillance programs and educational campaigns [4].

In addition to a very-low birth weight (<1500 g) and a very-low gestational age (<24 weeks); deficient oxygen regulation (fraction of inspired oxygen) is a powerful predictor factor for ROP and neonatal survival [5]. Despite advances in ROP treatment in centers with sufficient technology for ROP screening and excellent neonatal care, there is still lack of consensus regarding the oxygen level that could avoid retinal disease and still be considered safe for the neonate [6]. According to Chen et al. the need of oxygen varies at different developmental stages and phases of ROP: the use of low oxygen saturation (70–96%) during the first postnatal weeks and high oxygen saturation (94–99%) at postmenstrual ages of 32 weeks or older, were both associated with decreased risk for progression to severe stages of ROP [7].

Due to recent advances in neonatal intensive care and successful government screening programs, the incidence of the more advanced stages of ROP (stages 4 and 5) has been decreasing in developed countries over the past years [8].

The increasing body of peer-review evidence suggest that in low and middle-income countries, the neonatal care and ROP screening criteria are more variable. There is a tendency of severe ROP appearance in older and heavier babies (>32 gestational age [GA] and/or >1251 g of birth weight [BW] in moderated-poor developed countries [8–10]. Therefore, more mature babies may suffer from ROP stages, not typically found in these age groups, and must be examined despite being outside of the recommended guidelines for screening (birth weight ≤1500 g or gestational age of 30 weeks or less; newborns with a birth weight between 1500 and 2000 g or gestational age higher than 30 weeks, but under cardiorespiratory support or at risk for ROP; but still had record of have been exposed to high concentration of oxygen for long periods of time during neonatal care) [9, 10]. Moreover previously vascularized retinas may suffer regression and later reactivation of the disease, which force the retina specialist to implement longer surveillance periods and properly discriminate between normal and abnormal peripheral vascular changes on allegedly low risk ROP neonates [4].

In the following study, we will describe a wide array of peripheral vascular changes using fluorescein angiography in preterm neonates without high risk characteristics for ROP (more than 32 weeks of postmenstrual age and/or more than 1251 g birth weight) but with clinical history of have been exposed to high concentration of oxygen or have not been correctly monitored during neonatal care. All neonates developed clinical characteristics similar to ROP but with remarkably different patterns on fluorescein angiography (FA) and natural history than those already described. The vascular changes described herein also shares similarities with another clinical entity described in murine models of ROP known as oxygen-induced retinopathy (OIR) [11].

## Methods

Retrospective, two center, case series. The study was approved by the internal review boards of the “Asociación para Evitar la Ceguera en Mexico, IAP” and the Neonatal Intensive Care Unit of the “Monica Pretelini Saenz Hospital” hospitals in Mexico City and Toluca City respectively. The study was conducted according to the tenets of the declaration of Helsinki and all sensitive data were managed according to the Health insurance Portability and Accountability Act (HIPAA) rules. Due to the retrospective nature of the study, informed consents were not obtained at this time.

We reviewed all medical records and RetCam-III fundus and FA images (Clarity Medical Systems, Pleasanton, CA, USA) from two different hospitals. We included all files from diagnosed with ROP with a birth weight ≥1500 g or gestational age of ≥32 weeks, with fluorescein angiography performed. From each file, we extracted the gestational age, gender, birth weight, age at the first visit and history of supplemental oxygen, along with all medical notes regarding fundus description. Images files in JPEG format from the fundus examination and FA were extracted from the RetCam-II hard drive. Clinical description of the images and neonate assessment was done by a retinal specialist with extensive expertise in ROP (MAMC). Images were later classified according to the observed vascular abnormalities and findings were analyzed for similarities with ROP and OIR.

Descriptive statistics was done using an excel spreadsheet (Excel 2007; Microsoft Corp., Redmond, WA, USA) for means, standard deviations and standard error of the mean.

## Results

From a total of 294 infants diagnosed with ROP, we included 28 eyes from 14 patients with peripheral vascular abnormalities who met the inclusion criteria described in the “Methods” section. Mean gestational age was 33.7 ± 0.7 weeks (range 33–35 weeks); mean birth weight was 1848 ± 135 g (range 1650–2100 g) and mean age at first visit was 38.7 ± 1.8 weeks of postmenstrual age (range 38–43 weeks). Patient’s demographics are summarized on Table 1.

**Table 1:** Demographic characteristics of the 14 patients diagnosed with suspected OIR

Patient	Gestational age at birth (weeks)	Gender	Weight at birth (g)	Age at first visit (weeks)	History of supplemental oxygen
1	33	Female	1905	38	Yes
2	34	Male	1950	39	Yes
3	34	Male	1830	40	Yes
4	35	Male	2100	41	Yes
5	33	Female	1670	37	Yes
6	33	Female	1700	36	Yes
7	33	Male	1810	38	Yes
8	33	Female	1900	37	Yes
9	35	Female	1760	43	Yes
10	34	Male	1870	39	Yes
11	34	Male	1990	40	Yes
12	34	Male	2000	38	Yes
13	33	Male	1650	38	Yes
14	34	Female	1740	39	Yes

Image analysis showed two distinct type of peripheral vascular changes:

- *Group 1 or non*-*proliferative*; the hallmark were extensive areas of capillary non-perfusion along with widespread arteriovenous shunting between adjacent primary vessels, tortuosity of primary vessels (“plus-like” disease), abnormal budding of tertiary vessels and capillaries, abnormal capillary tuffs and absence of foveal avascular zone. Vascular changes were readily identified in FA images (cases 1, 2 and 3).
- *Group 2 or proliferative*; the hallmark was some or all the characteristic of group 1 plus leakage of dye from the boundary between perfused and non-perfused retina and/or optic disc (case 4).

## Representative cases

### Case #1

**Fig. 1:**
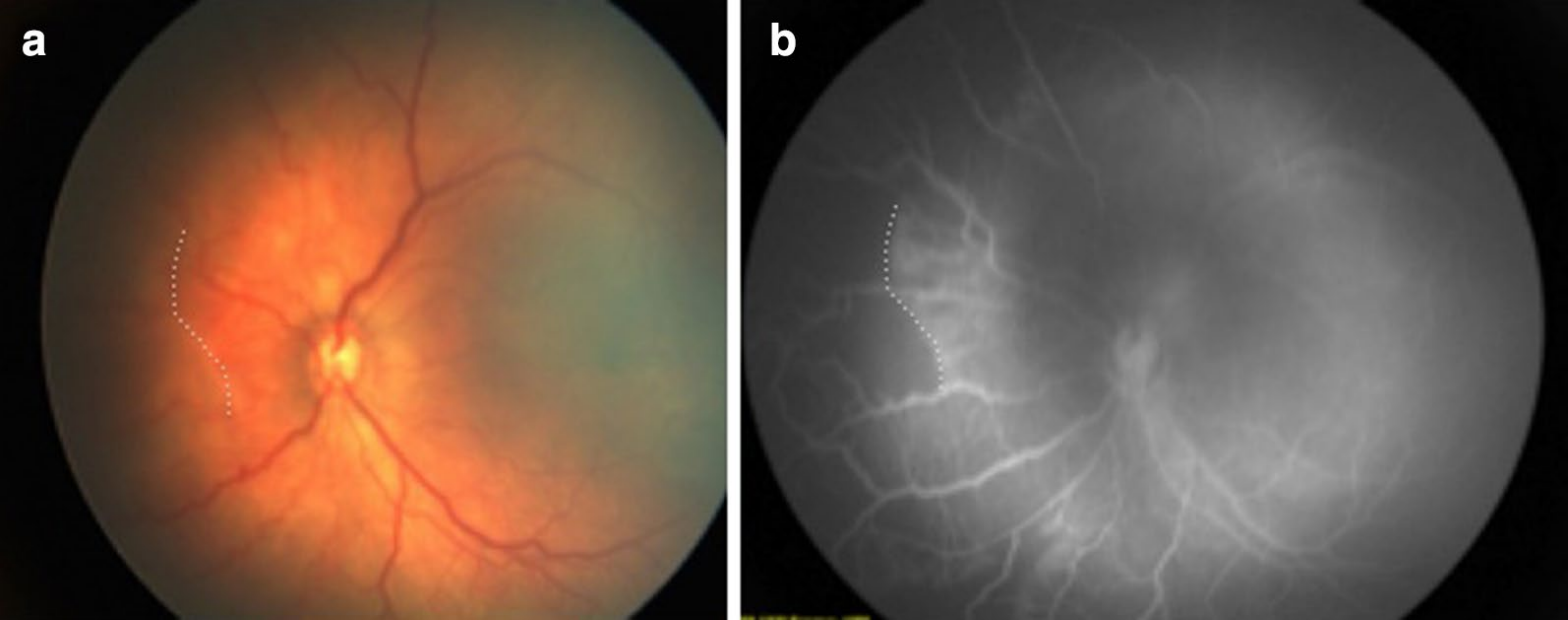
Color picture (**a**) and FA (**b**) show areas of retinal non-perfusion with vasoobliteration and arteriovenous shunting within areas of the retina that are already vascularized (*white dashed line at* the limit of vascularized and non vascularized). The borders of this area are irregular, and there are no sprouts visible. It is noteworthy that there is absence of foveal avascular zone

Patient with a non-proliferative OIR (group 1) of 33 weeks of GA and 1650 g of birth weight examined at 38 weeks of corrected age (Fig. 1a, b).

### Case #2

**Fig. 2:**
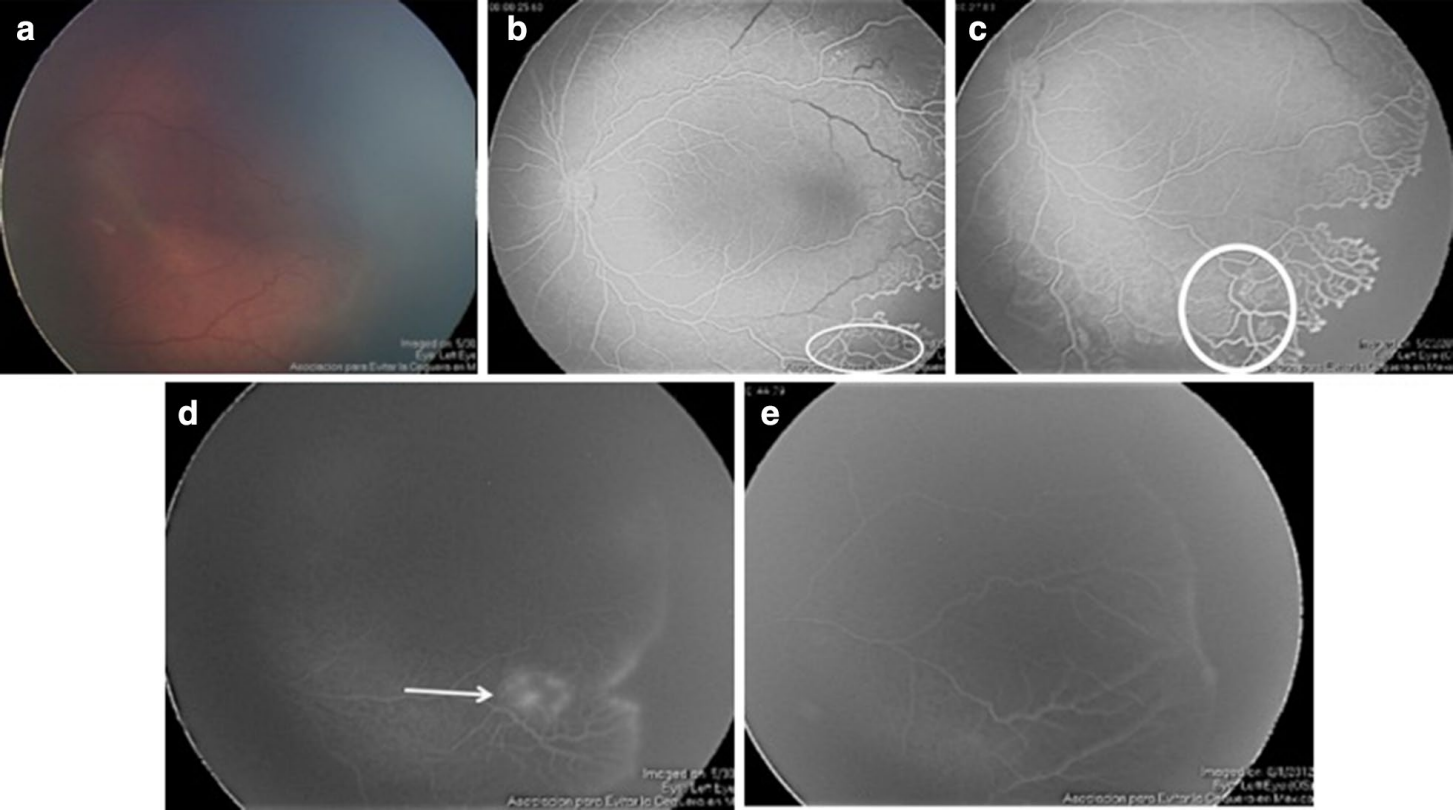
Fundus examination showed a flat demarcation-like line in retinal periphery (**a**). FA images showed areas of capillary non-perfusion and arteriovenous shunting in what appeared to be mature retinal vessels (**b**, **c**). The patient was placed on rigorous monitoring and intensive regulation of oxygen. She was reassessed 1 week later (38 weeks). Fundus examination revealed formation of new vessels and patches (“islands”) of leaking vessels (**d**). Twelve weeks later, weekly screening showed that vessels continued growing toward the periphery (**e**). This patient was classified as group 1

Female neonate of 33 weeks of gestational age at birth, birth weight of 1900 g and history of have been exposed to high concentration of supplemental oxygen. The patient was screened for ROP at 37 weeks of gestational age (Fig. 2).

### Case #3

**Fig. 3:**
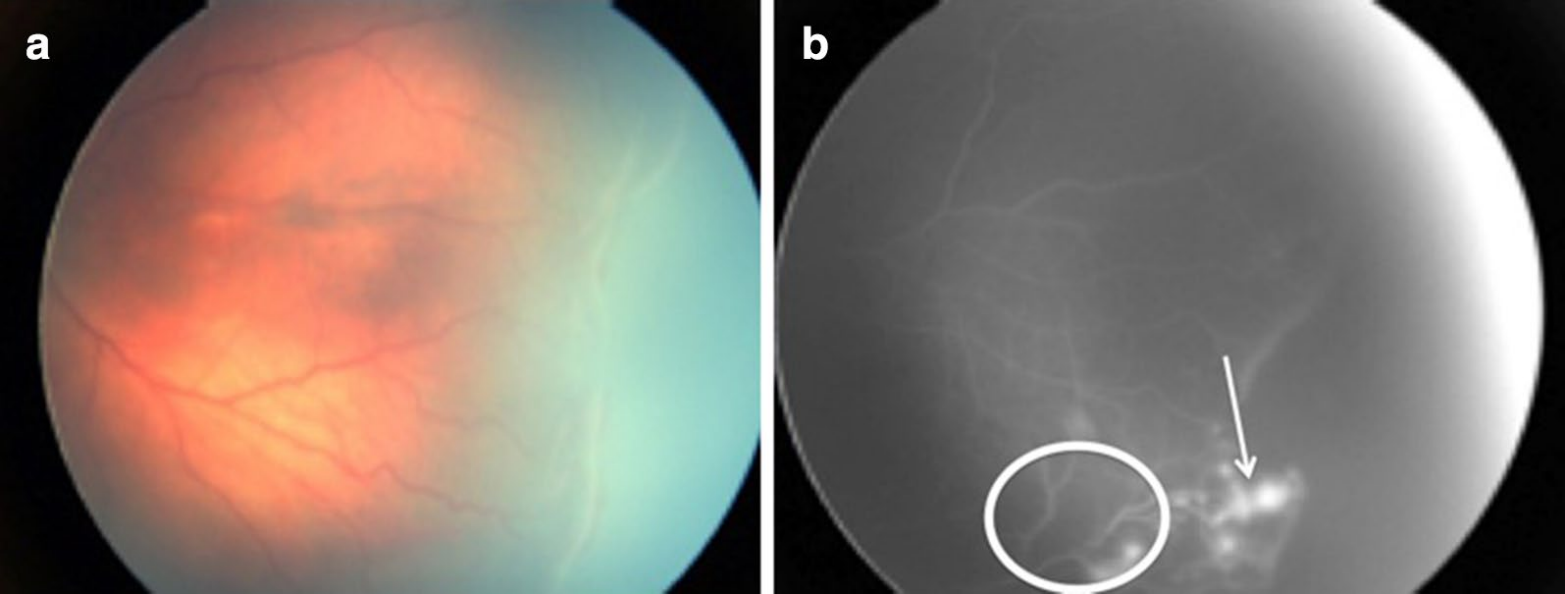
Image **a** shows an elevated demarcation line. Image **b** shows microaneurysms or tufts posterior to the ridge along with discrete leakage of fluorescein at the ridge (*white arrow*). There are also vasoobliterative and capillary free areas (*circle*). Of note is that the demarcation line is irregular and we can find capillary free islands posterior to the ridge

Group 1 OIR of a newborn of 33 weeks of gestational age and 1905 g at birth examined at 38 weeks of corrected age (Fig. 3).

### Case #4

**Fig. 4:**
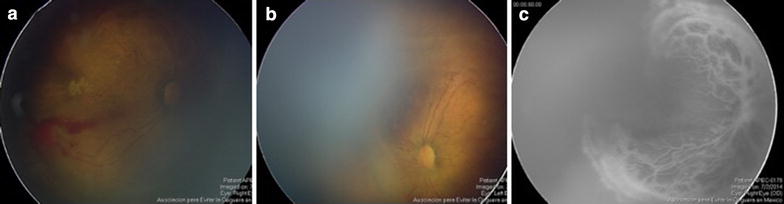
Fundus examination showed neovascularization in zone 1 (along temporal vascular arcades), extensive areas of capillary drop-out, subhyaloid and vitreous hemorrhages and macular fibrosis (**a**, **b**). FA of right eye shows vascular tortuosity and leakage secondary to neovascularization (**c**). The patient was classified as group 2

Male neonate of 34 weeks of gestational age, birth weight of 1870 g and history of have been exposed to high concentration supplemental oxygen including tracheal intubation. He had sepsis during hospitalization (Fig. 4).

## Discussion

Immature retinas are especially susceptible to damage during development. When compared to adults, choroidal flow is almost absent and retinal blood flow has a narrow flow range that must be strictly auto-regulated. Moreover, retinas in neonates are rich in mitochondria; capable of producing high amounts of reactive oxygen species, which cannot be counterbalanced by antioxidants in utero. Consequently, excessive supplemental oxygen, a high fluctuation of oxygen saturation in the retina (hypoxia-hyperoxia) and a strictly choroidal blood flow, have a crucial role in the pathogenesis of proliferative vascular diseases in premature newborns [12–17].

In our country most of the Neonatal Intensive Care Units do not count with blenders that allow continuing oxygen regulation when hospitalized or discharged for parental control of oxygen intake. Consequently, we do not have a record that states the level of oxygen received at any time during the day over the time-lapse that oxygen is administered. Recently, an important publication from our country stated that uncontrolled oxygen supplementation is the major risk for ROP requiring treatment for infants born >32 weeks of GA, and that its regulation diminished the frequency of treatment for ROP [18]. This is the importance of our paper, that positions that there are clear angiographic differences among those with low-risk for developing ROP from regular risk ROP patients; findings that support that there must be a different etiology for the development of the retinopathy, which is still needed to be confirmed with histology and molecular studies.

In middle-income countries, neonatal survival has been increasing in later years. Which brings as a consequence, an increase in neonatal care-related pathologies, including ROP [19]. Standard of care in neonatal intensive care units (oxygen not well regulated), surveillance guidelines and treatment can vary widely in these countries; which leads to increase in ROP-like disease events, even in heavier and more mature babies, not typically considered at risk for ROP in developed countries. Zepeda-Romero et al. reported a case of a newborn of 1280 g birth weight who presented arteriovenous shunts capillary loss and multiple areas of non-perfusion; similar to the cases described herein. In this case series, we describe the peripheral vascular abnormalities of 28 eye belonging to 14 neonates with no medical history of extreme prematurity or very low birth weight, but who have been exposed to unmonitored high concentration of oxygen for variable periods of time [20]. All eyes developed vascular abnormalities, similar to those described as typical ROP but with distinctive differences like venous beading, tortuosity and dilation of major retinal vessels and capillaries (“plus-like” disease), arteriovenous shunting, areas of capillary closure, abnormal capillary tufts, microaneurysms, patches (“islands”) of leaking vessels. An additional finding is the absence of avascular foveal zone which has been reported by Henaine et al. [21] in 50% of patients born at 36 weeks of gestational age or more.

In patients with ROP, newly formed vessels are not fully mature, resulting in areas of non-perfusion. Although this vascular abnormality is not usually seen in patients with more mature retinas, it is a key component of murine models of ROP known as OIR [13, 14, 16, 17].

**Fig. 5:**
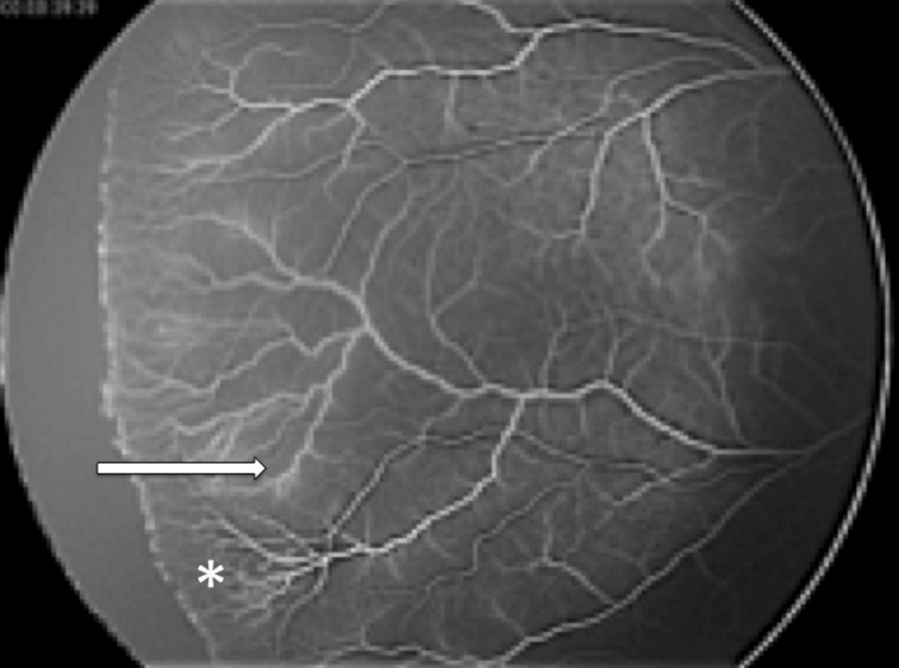
Leakage of dye in immature vessels in a 28 weeks of gestation baby with a ROP stage 1

The hallmark of ROP stage 1 is a visible flat demarcation line between vascular and avascular peripheral retina [22]. In our case series, patients classified as having vascular abnormalities belonging to group 1, also had a flat demarcation line. But, unlike ROP, a clear capillary-free area, posterior to the demarcation line, can also be observed. This capillary-free zone also shows arteriovenous shunting among primary vessels, not typically seen in ROP stage 1. Additionally, patients with ROP stage 1 show perivascular leakage of dye during FA; a characteristic not found in patient classified as group 1 in this case series. The authors speculate that this phenomenon could be explained because in neonates with ROP, the cellular junctions in immature retinal vessels are still developing and the vessels are still not fully supported by pericytes (Fig. 5). Therefore, leakage could be observed. On the contrary, neonates in group 1 had no clear history of extreme prematurity, which make highly probable that they had more developed retinas with more mature vessels, already fully supported by pericytes which do not allowed leakage. Obliteration of previously formed retinal vessels, resulting in extensive areas of capillary drop-out could also be observed in patients from group 1.

Stage 2 ROP is characterized by an elevated ridge at the demarcation line which subtly leaks fluorescein at the forming ridge. In our series, patients in group 1 who presented a ridge, also presented leakage but to a lesser degree, possibly indicating more mature vessels. Vascular abnormalities beyond the posterior limit of the ridge are not classically observed in stage 2 ROP [23]. To our knowledge, there is only one report of vascular tufts posterior to the ridge adjacent to retinal hypoperfusion areas. In our series, patients typically shown aneurysm or tufts in an “island” pattern within normal-looking retina, far posterior to the demarcation line and ridge. Conversely to ROP cases, patients within group 1 could also show a marked boundary, easily distinguishable between vascular and avascular areas of the retina and areas of capillary non-perfusion. The demarcation line had a “dentate” pattern (deep bays) within an irregular ridge.

In stage 3 ROP, neovascularization appears at the ridge. In our series, patients classified as group 2 or proliferative cases, also had neovascularization at the optic disc. This feature is very uncommon in ROP. Finally, the most aggressive cases in group 2 also shown a “4-lobe” vascular topography, similarly to cases with Aggressive posterior retinopathy of prematurity (APROP) [24].

## Conclusion

In summary, this case series describes the peripheral vascular abnormalities in a group of patient older than 32 weeks of gestational age, and heavier than 1500 g but who developed specific morphological and functional vascular angiographic findings, despite having a more mature appearance in their retinal vasculature. The good amount of differences with classic ROP on fundus examination and FA, makes the authors wonder if there are additional pathological process in play besides the already described for ROP. At this time, the lack of evidence prevent us to label this vascular abnormalities as solely induced by oxygen dysregulation at the neonatal intensive care unit (like OIR in the laboratory) or as a different manifestation of ROP within the same spectrum of the disease, in heavier and more mature neonates. A complete ophthalmological examination (including fundus examination and FA evaluation) is advisable in all neonates, even if they are older of 32 weeks of gestational age and/or heavier than 1500 g, whenever they have history of have been exposed to excessive oxygen; in order to make precise diagnosis and establish proper treatment if needed. More studies are needed in order to identify the precise pathologic mechanism and the entire spectrum of peripheral vascular abnormalities in neonates without strict criteria of prematurity, but that are still being exposed to high concentrations of oxygen during neonatal care.

## Abbreviations

OIR: oxygen induced retinopathy
ROP: retinopathy of prematurity
GA: gestational age
BW: birth weight
FA: fluorescein angiography
HIPAA: Health insurance Portability and Accountability Act
MAMC: Maria Ana Martínez Castellanos
APROP: aggressive posterior retinopathy of prematurity

## References

[1] Hellström A, Smith LEH, Dammann O (2013). Retinopathy of prematurity. Lancet.

[2] Wright KW, Sami D, Thompson L (2006). A physiologic reduced oxygen protocol decreases the incidence of threshold retinopathy of prematurity. Trans Am Ophthalmol Soc.

[3] Owen LA, Hartnett E (2014). Current concepts of oxygen management in retinopathy of prematurity. J Ophthalmic Vis Res.

[4] Fielder A, Blencowe H, O’Connor A, Gilbert C (2015). Impact of retinopathy of prematurity on ocular structures and visual functions. Arch Dis Child Fetal Neonatal Ed.

[5] Cavallaro G, Filipi L, Bagnoli P (2014). The pathophysiology of retinopathy of prematurity: an update of previous and recent knowledge. Acta Ophthalmol.

[6] Hartnett ME, Lane RH (2013). Effects of oxygen on the development and severity of retinopathy of prematurity. JAAPOS.

[7] Chen ML, Guo L, Smith LE, Dammann CE, Dammann O (2010). High or low oxygen saturation and severe retinopathy of prematurity: a meta-analysis. Pediatrics.

[8] Gilbert C, Fielder A, Gordillo L (2005). Characteristics of infants with severe retinopathy of prematurity in countries with low, moderate and high levels of development: implications for screening programs. Pediatrics.

[9] American Academy of Pediatrics (2013). Screening examination of premature infants for retinopathy of prematurity. Pediatrics.

[10] Good WV, Early Treatment for Retinopathy of Prematurity Cooperative Group (ETDRS) (2004). Final results of the early treatment for retinopathy of prematurity (ETROP) randomized trial. Trans Am Ophthalmol Soc.

[11] Dembinska O, Marina-Rojas L, Chemtob S (2002). Evidence for brief period of enhanced oxygen susceptibility in the rat model of oxygen induced retinopathy. IOVS.

[12] Prezemyslaw S, Joyal JS, Rivera JC (2010). Retinopathy of prematurity: understanding ischemic retinal vasculopathies at an extreme of life. J Clin Invest.

[13] York JR, Landers S, Kirby RS (2004). Arterial oxygen fluctuation and retinopathy of prematurity in very low birth weight infants. J Perinatol.

[14] Penn JS, Henry MM, Tolman BL (1994). Exposure to alternating hypoxia and hyperoxia causes severe proliferative retinopathy in the newborn rat. Pediatr Res.

[15] O’Bryhim BE, Radel J, Macdonald SJ (2012). The genetic control of avascular area in mouse oxygen-induced retinopathy. Mol Vis.

[16] Roberts R, Zhang W, Ito Y (2003). Spatial pattern and temporal evolution of retinal oxygenation response in oxygen-induced retinopathy. Invest Ophthalmol Vis Sci.

[17] Shao Zhuo, Dorfman Allison L (2011). Choroidal involution is a key component of oxygen-induced retinopathy. Invest Ophthalmol Vis Sci.

[18] Zepeda-Romero LC, Lundgren P, Gutiérrez-Padilla JA (2016). Oxygen monitoring reduces the risk for retinopathy of prematurity in a Mexican population. Neonatology.

[19] Visser L, Singh R, Young M, ROP Working Group, South Africa (2013). Guideline for the prevention, screening and treatment of retinopathy of prematurity (ROP). S Afr Med J.

[20] Zepeda-Romero LC, Oregon-Miranda AA, Lizárraga-Barrón DS (2013). Early retinopathy of prematurity findings identified with fluorescein angiography. Graefes Arch Clin Exp Ophthalmol.

[21] Henaine-Berra A, Garcia-Aguirre G, Quiroz-Mercado H (2014). Retinal fluorescein angiographic changes following intravitreal anti-VEGF therapy. JAAPOS.

[22] Preslan MW, Butler J (1994). Regression pattern in retinopathy of prematurity. J Pediatr Ophthalmol Strabismus.

[23] Ng EYJ, Lanigan B, O’Keffe M (2006). Fundus fluorescein angiography in the screening and management of retinopathy of prematurity. J Pediatr Ophthalmol Strabismus.

[24] Yokoi T, Hiraoka M, Miyamoto M (2009). Vascular abnormalities in aggressive posterior retinopathy of prematurity detected by fluorescein angiography. Ophthalmology.

